# What Will Cure a Cough

**Published:** 1886-02

**Authors:** 


### WHAT WILL CURE A COUGH.

A cough is a work of intelligence of a current of electricity in a wind pipe. We don't want any mistake made about what we mean; an intelligent work of a current of electricity is performed in a cough. A cough is actually an attempt of a current of this almighty influence to cast off a gathering of gas and water on the surface of the wind pipe. The cough is only a spurting of a current of an almighty out of a chest with such violence as to throw out all the unnatural encumbrance on the pipe. When a cough is made a contraction of the whole chest is made—just to the extent that a current of this worker is discharged by the cough.

The brain is not operated in this work. It often attempts to stop it, but it is a poor attempt, for it will most always fail, We are always in our senses when a brain is operated for an intelligent work. A cough is performed for a minute and sometimes for hours without consciousness on our part.

Mucous is a saturation of the water of a pipe by a gas from the stomach. It is but gas under water. A chance is offered in this understanding of the substance to obtain a remedy for a cough. A thousand doctors would see the remedy if it was known what mucous was.

Anything that will stop the generation of gas in the stomach will stop a cough. When a person is sick with a cold the stomach is sure to be out of order. The bad stomach permits a gas to be created. It can always be smelled. No cough is possible without a mucous in a pipe, or pus, and a mucous is impossible in a pipe without a gas from a stomach. Every cough other than that produced by a decomposition of lung, or bronchial pipe, is only a tickling of gas and water on a wind pipe.

What is capable of stopping the creation of gas? A good condition of stomach is sure to stop it.

A glass of soda water is the thing that will prevent a construction of mucous. It is all that is necessary, except to open a closed pore if it is not opened, and it is capable of curing a cough, and preventing a cold continuing. Let our patrons try it. The gas discharged from a bad stomach passes through the nostrils and mouth. When a breath is inspired some of the gas is drawn into the wind and all the pipes of the chest and mixed with the water in the pipes. If the gas is discharged for a great length of time it will create a sore nose and what are called canker or cold sores on the mouth and lips. These sores are only a poisoned tissue. A cough will sometimes continue as a result of a cold or closing of the pores of the body for months, or weeks, and finally give a corrosion to the bronchial pipes and lung. When this is the character of a cough, a constant disorganization of the process of digestion is possessed. Just the same condition of disordered digestion as was first produced by the cold. An opening of pores is still wanted, and it must be preformed.—*Problems of Nature.*

